# Lack of associations of microRNAs with severe NAFLD in people living with HIV: discovery case-control study

**DOI:** 10.3389/fendo.2023.1230046

**Published:** 2023-09-22

**Authors:** Mario Frías, Diana Corona-Mata, Jose M. Moyano, Angela Camacho-Espejo, Pedro López-López, Javier Caballero-Gómez, Inmaculada Ruiz-Cáceres, María Casares-Jiménez, Ignacio Pérez-Valero, Antonio Rivero-Juárez, Antonio Rivero

**Affiliations:** ^1^ Unit of Infectious Diseases, Hospital Universitario Reina Sofia, Clinical Virology and Zoonoses, Maimonides Biomedical Research Institute of Cordoba, University of Córdoba, Córdoba, Spain; ^2^ CIBER de Enfermedades Infecciosas, Instituto de Salud Carlos III (CIBERINFEC, ISCIII), Madrid, Spain; ^3^ Animal Health Department, Animal Health and Zoonoses Research Group (GISAZ), University of Córdoba, Córdoba, Spain; ^4^ Department of Computer Science and Artificial Intelligence, Andalusian Research Institute in Data Science and Computational Intelligence (DaSCI), University of Granada, Granada, Spain

**Keywords:** microRNA, HIV, NAFLD, CAP, PLWH, steatosis

## Abstract

**Background & objective:**

Nonalcoholic fatty liver disease (NAFLD) is highly prevalent in people living with HIV (PLWH) and the expression of some microRNAs could be useful as biomarkers for the diagnosis of NAFLD. The aim of this study was to identify patterns of differential expression of microRNAs in PLWH and assess their diagnostic value for NALFD.

**Methods:**

A discovery case-control study with PLWH was carried out. The expression of miRNAs was determined using HTG EdgeSeq technology. Cases were defined as patients with severe NAFLD and controls as patients without NAFLD, characterized using the controlled attenuation parameter (CAP). Cases and controls were matched 1:1 for age, sex, BMI, CD4+ lymphocyte count, active HCV infection, and ART regimen.

**Results:**

Serum 2,083 simultaneous microRNA transcripts were analyzed using HTG technology and compared between cases and controls. Forty-five patients, 23 cases, and 22 controls were included in the study. In the analysis of the expression pattern of the 2,083 microRNAs, no differential expression patterns were found between both groups of patients included in the study.

**Conclusion:**

Analysis of the microRNA transcriptome profile of nonobese PLWH with severe NAFLD did not appear to differ from that of patients without NAFLD. Thus, microRNA might not serve as a proper biomarker for predicting severe NALFD in this population.

## 1 Introduction

Nonalcoholic fatty liver disease (NAFLD) is defined as the accumulation of fat in hepatocytes that is unrelated to severe alcohol consumption (1). Simple steatosis can progress to steatohepatitis and the appearance of liver fibrosis and hepatocellular carcinoma (HCC) (2). Therefore, patients with NAFLD have a higher risk of mortality from hepatic causes, as well as cardiovascular events (3, 4). It is estimated that the global prevalence is 25% in the adult population (5), and the global incidence of NAFLD ranges from 28 to 52 cases per 1,000 people/year (6), making it presumable that a large proportion of cardiovascular events, HCC, and deaths from liver disease will be related to NAFLD in the coming years. The most important factor for the development of NAFLD is obesity (7); however, there are other factors that have been shown to be associated with this pathology (8) and can serve as biomarkers.

The prevalence of NAFLD in people living with HIV (PLWH) is more frequent than in the general population, being approximately 34% according to a recent meta-analysis (9). In addition, PLWH with NAFLD have a higher prevalence of steatohepatitis development, as well as greater liver function impairment compared to HIV-uninfected NAFLD patients (10). In addition to factors inherent to HIV infection itself, numerous recent studies have reported weight gain in PLWH who switched from older antiretroviral therapy (ART) to integrase strand transfer inhibitor-based regimens (11, 12). For all these reasons, studies are needed to address the potential risk factors for the development and identification of biomarkers that could serve as diagnostic approaches for NALFD in this special population.

The gold-standard method for the diagnosis of NAFLD is the histological study of liver biopsy because it allows distinguishing between simple steatosis and steatohepatitis (13). However, due to its invasive nature, liver biopsy is not a useful tool for epidemiological screening or as a follow-up method (14). The controlled attenuation parameter (CAP), using Fibroscan^®^ (Echosens; Paris, France), was introduced as a new technique to detect fatty liver disease. This method is a non-invasive technique, has low inter- and intraobserver variability (15), and provides a numerical value that is highly correlated with the histological grade of NAFLD (16). However, the measurement by CAP has some limitations, since diagnostic performance decreases in the most severe cases of NAFLD and the correlation of CAP and liver biopsies in obese patients (Body mass index; BMI > 30 kg/m^2^) is lower (16). This highlights the need to identify new alternatives for the diagnosis of NAFLD. In recent years, new biomarkers with diagnostic potential for NAFLD have emerged, such as microRNAs (miRNAs), since they participate in the regulation of lipid metabolism, the development of obesity, the regulation of glucose homeostasis, and the pathogenesis of steatohepatitis (17). Different studies have found a relationship between the appearance of NAFLD and alterations in the expression levels of different miRNAs (18). Therefore, circulating miRNAs could be highly relevant biomarkers for the diagnosis and prognosis of NAFLD (17). Thus, miR-122, miR-34a and miR-192 have been identified as potential markers for NAFLD. In the PLWH population, only miR-200a has been suggested as a NAFLD biomarker, showing possible applicability to predict the progression of NAFLD and liver damage in this population (19). Nevertheless, wide evaluation of the whole spectrum of microRNAs as potential biomarkers in PLWH is lacking. Therefore, the aim of our study was to identify miRNA expression profiles associated with the presence of severe NAFLD in the PLWH population.

## 2 Materials and methods

### 2.1 Study design and selection of case-control patients

We designed a case-control study carried out in a cohort of PLWH under follow-up in the Infectious Diseases unit of the Reina Sofía University Hospital in Córdoba (Spain) between January 2012 and December 2018. All patients included in this cohort had a valid baseline determination of CAP, defined as a measurement with a success rate greater than or equal to 60% and an IQR less than or equal to 30% (20, 21). The CAP value was measured in each patient by means of transient elastography (TE) using a 3.5 MHz M probe (FibroScan, Echosens; Paris, France) after at least 4 hours of fasting. The test was performed by qualified technicians following the standard technique (22). The cutoff points for the classification of steatosis according to the CAP value were those established in the meta-analysis by Karlas and colleagues (16). Briefly, a CAP value less than 248 dB/m was considered the total absence of liver steatosis (S0), and a CAP value greater than 280 dB/m was considered severe steatosis (S3).

Cases and controls were matched 1:1 for age, sex, BMI, CD4^+^ lymphocyte count, active HCV infection, and ART regimen. The inclusion criteria were: i) HIV infection, ii) being on ART, and iii) undetectable serum HIV viral load in the last 12 months. The exclusion criteria were: i) hepatitis B virus (HBV) infection, determined by the presence of surface antigen (HBAgS); ii) alcohol consumption equal to or greater than 20 grams/day; iii) BMI higher than 30 kg/m^2^; iv) liver cirrhosis; v) diabetes mellitus; and vi) use of lipid-lowering treatments.

### 2.2 Variables collected

Variables collected included age (expressed in years), sex, BMI (expressed as kg/m^2^), active HCV infection, and ART regimen. In addition, the percentage of absolute CD4^+^ lymphocytes (cells/mL), nadir CD4^+^ lymphocyte count (cells/mL), HIV viral load (IU/mL), and time with viral suppression of HIV (expressed in months) were also recorded.

From all patients, a 500 µL serum sample was collected at baseline and cryopreserved at -80°C in the Andalusian Health System Biobank (National Register Reference: B.0001601).

### 2.3 Determination of the expression of miRNAs

The expression of miRNAs was determined using HTG EdgeSeq technology (HTG Molecular Diagnostics, Inc., Tucson, AZ). The HTG EdgeSeq system combines HTG’s proprietary quantitative nuclease protection assay (qNPA) chemistry with a Next Generation Sequencing (NGS) platform to enable the semiquantitative analysis of a panel of targeted genes in a single assay. qNPA chemistry does not require nucleic acid extraction from serum samples. Samples were processed in accordance with OP-00034, HTG EdgeSeq Processing (Instrument Method).

To study the expression of miRNAs, the miRNA Whole Transcriptome Assay, Rev. 3 (23-August 2018) panel was used, in which the expression of 2,083 simultaneous miRNA transcripts was quantitatively measured. The analysis of the miRNAs was performed from 15 µL of serum using the HTG EdgeSeq processor. First, qNPA was used to identify and measure miRNA gene expression. The output of qNPA is protection probes corresponding to the expressed target miRNA. The results of the qNPA technique were subjected to PCR to amplify the material obtained using a pair of specific primers with barcoding for each patient. Once the amplification was carried out, the library was purified to subsequently perform quantitative PCR (qPCR) and determine the concentration of probes in each patient.

Samples were subjected to a postsequencing HTG technology quality control statistical test. Quality control is based on sequencing of ANT (negative process control) added to each sample. The raw data obtained from sequencing were normalized by transforming the counts by fitting the total reads within each sample. Once the quantification was carried out, the library was normalized to ensure that the samples had a homogeneous input concentration to the sequencer. For this, the data obtained from the quantification were entered into the HTG EdgeSeq Library Calculator. Sequencing was performed using the Illumina MiSeq^®^ sequencer with sequencing-by-synthesis (SBS) technology, which uses *in vitro* clonal amplification by bridging PCR. Sequencing results were generated in FASTQ files, which were loaded into HTG Parser Software. The HTG EdgeSeq host software was used to align the FASTQ files to the probe list to collate the data. Data are provided as a data table of raw, QC raw, CPM (counts per million), and median normalized (available in **Supplemental Data**).

### 2.4 Statistical analysis

The minimum sample size was calculated in a 1:1 ratio to ensure that significant differences in miRNA expression were obtained. For this purpose, a frequency of expression of each of the miRNAs of 10% in controls and 50% in cases, a statistical power of 80% and a safety level of 95% were considered. With these considerations, the minimum sample size was 38 patients (19 cases and 19 controls). Categorical variables are presented as absolute (number of cases [n]) and relative frequencies (%), while continuous variables are presented as medians (interquartile range [IQR]). The Mann-Whitney U test was performed to compare medians between the case and control patient groups.

### 2.5 Data mining analysis

Given the large number of miRNA attributes, to make the study feasible, feature selection using the Lasso technique was performed (23), considering the group (case/control) variable as the objective. We also built different classification methods using machine algorithms such as logistic regression, random forest, SVM, and gradient boosting (24), where the miRNAs were used as input features and the group was used as the target. In order to determine the effect of certain specific features such as sex and HCV infection, similar sub-analyses were conducted: on the one hand, the same process is carried out but considering only male and female patients separately; on the other hand, patients with and without previous HCV infection are also separated to be analyzed.

### 2.6 Ethical aspects of the study

This study was designed and conducted in accordance with the Declaration of Helsinki. All patients signed informed consent that authorized the storage and processing of samples in routine diagnostic processes by the Biobank of the Public Health System of Andalusia. The study protocol was approved by the CEIC (Clinical Trials and Ethics Committee) with code PI18/00606.

## 3 Results

### 3.1 Patients included

Selection criteria were met by 379 patients. Of these, 45 patients (23 cases and 22 controls) were selected, achieving the minimum sample size needed. The main clinical characteristics of patients are shown in **Table 1**, where the baseline characteristics between cases and controls are compared to verify the correct matching between them. A 40% and 43.5% of patients had active HCV infection in controls and cases, respectively. The only statistically significant variable was the defining case-control variable (CAP value).

**Table 1:** Main patient characteristics.

Clinical variable	Non-NALFD (controls) N = 22	Severe NAFLD (cases) N = 23	p
BMI, kg/m^2^ (IQR)	25.1 (22.6-26.56)	24.5 (22.1-26.6)	0.785
CD4^+^ count cell, cel/mL (IQR)	572 (440-849)	592 (421-732)	0.594
Age, years (IQR)	48.5 (41.7-51.5)	47.5 (44.7-52.2)	0.724
CAP, dB/m (IQR)	214 (186-232)	306 (289-337)	<0.001
Liver stiffness, kPa (IQR)	5 (4.3-8.4)	5.9 (4.7-8.9)	0.207
Sex, male (%)	19 (86.4)	20 (87)	0.953
Active HCV infection, n (%)	9 (40)	10 (43.5)	0.862
ART third drug, n (%)			0.856
NRTI	11 (50)	11 (47.8)	
Protease inhibitor	10 (45.5)	10 (43.5)	
Integrase inhibitor	1 (4.5)	2 (8.7)	

BMI, body mass index; CAP, controlled attenuated parameter; HCV, hepatitis C virus; ART, antiretroviral therapy; NNRTI, nucleoside/nucleotide reverse transcriptase inhibitors.

### 3.2 Statistical quality control of HTG technology

The graphical representation of the statistical process control and the quality control chart for ANT is shown in **Figure S1**, which shows that only one of the case samples (EHep-03-01; marked with a red dot) did not meet the required quality control criteria and was eliminated for normalization and exploratory analysis. **Figure S2** shows the correlation between all RNA samples processed in this study and reference RNA samples (human brain).

### 3.3 Exploratory analysis

**Figure 1:**
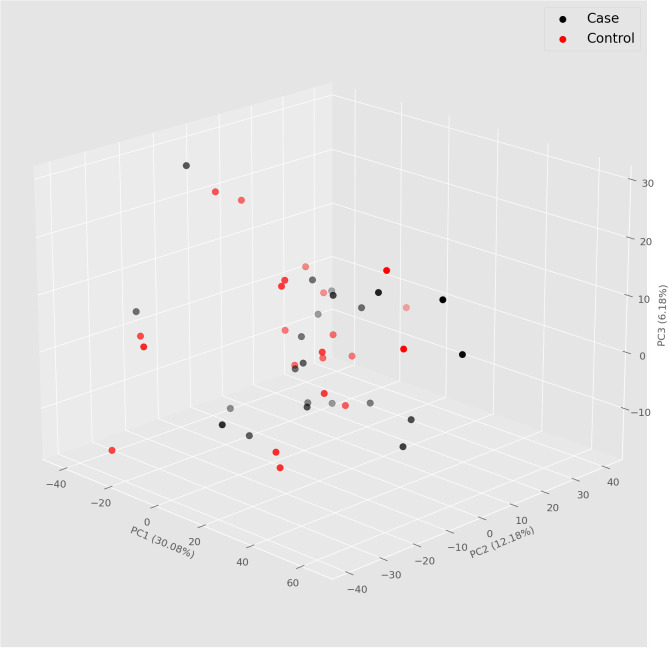
Principal component analysis (PCA) 3D plot showing the clustering in two groups of patients according to case and control.

Dimensional reduction by principal component analysis revealed that both cases and controls are closely related in terms of their expression pattern of serum miRNAs (**Figure 1**).

**Figure 2:**
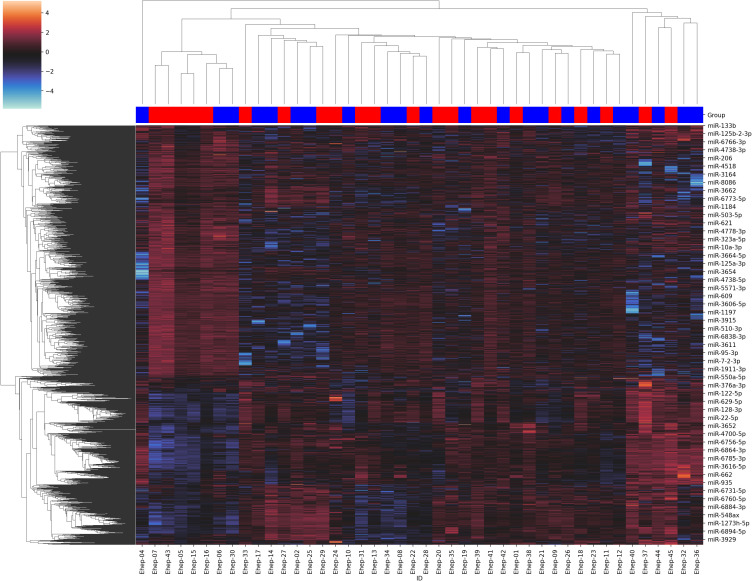
Heatmap and clustering of the normalized data. The cases are in blue and the controls are in red.

The exploratory analysis generated from the 44 study samples measured the expression of each miRNA, obtaining the raw data of the amount of expression of the miRNAs as count per million (CPM). Subsequently, the data obtained were normalized by means of transformation by the median, thus obtaining the normalized data (**Supplemental Data**). These already normalized expression data are represented in the heatmap using the Z scale (**Figure 2**). This heatmap revealed no differences in the expression pattern of miRNAs between case (blue) and control (red) patient group.

### 3.4 Data mining

By means of the Lasso technique, the set of miRNA attributes was reduced to only 29, with the rest considered irrelevant. From this set of 29 attributes, the possible univariate correlation between each of them and the objective variable was calculated; no significant correlations were obtained in any case between any of the attributes and the group. Despite these poor univariate correlations, a second study aiming to obtain higher-level correlations among the 29 selected variables jointly and the group was performed through machine learning algorithms. These models obtained AUROC values between 0.5 and 0.61, demonstrating very poor performance; this indicates that no clear high-level correlations among the miRNA attributes and the patient group exist, since the classification methods were unable to correctly model such relationships. It should be noted that sub-analyses involving patients separated by sex and previous HCV infection yielded similar results, i.e., strong relationships are not found even when analyzing the patients by these groups independently.

## 4 Discussion

Our study does not show differences in the expression pattern of serum miRNAs in PLWH patients who could be associated with the presence of NAFLD. Analysis of the normalized data did not reveal any pattern of differential expression of miRNAs between patients who had severe NAFLD and those who did not. Therefore, our results suggest that serum miRNAs are not useful as biomarkers for the diagnosis of severe NAFLD in PLWH patients.

Although most of the studies that analyze the expression profiles of miRNAs in NAFLD have been carried out in the general population, the results of this study are contrary to what has been reported regarding some miRNAs linked to NAFLD (18, 25–27). However, these studies have great methodological heterogeneity among themselves. One of the most important methodological differences lies in the normalization of the data. In some of these studies, this normalization is inadequate or not even carried out; therefore, it can lead to biased conclusions. In this sense, data normalization is crucial in studies where miRNA expression profiles are analyzed (28, 29). However, to date, there is no consensus for the process of standardization of the results, and this fact could significantly hinder the identification of miRNAs that may be useful as biomarkers and reproduced between different studies. In addition, other factors may influence the heterogeneity of results, such as sample processing, RNA extraction, and the technology used to determine the different miRNA expression profiles.

Due to the previously mentioned methodological heterogeneity of the studies, the meta-analysis carried out by Liu and colleagues is a study that includes all the studies that have analyzed miRNAs in the general population with the aim of identifying the potential usefulness of miRNAs as NAFLD biomarkers (18). Papers were included based on the diagnostic accuracy of NAFLD. Thus, the meta-analysis only included studies that reported data regarding the ability of certain miRNAs to diagnose NAFLD, as well as steatohepatitis. The manuscript highlighted miR-122 and miR-34a as the miRNAs with the greatest diagnostic capacity for NAFLD and steatohepatitis, with areas under the curve of 0.82 and 0.78, respectively. However, one of the main limitations of this meta-analysis is the fact that it includes studies whose analyses have not been adjusted for BMI, with being overweight possibly the greatest risk factor for the development of NAFLD (30, 31). An example of this limitation can be seen in the studies that highlight these two miRNAs as potential biomarkers for the diagnosis of NAFLD (26, 27, 32, 33). In these studies, differences in the expression profiles of miR-122 and miR-34a were observed between patients with NAFLD and healthy controls. The main limitation of these studies is that BMI was significantly higher in the NAFLD group. Therefore, the design of these studies does not allow us to conclude that the differential pattern of these miRNAs is associated with NAFLD, since they are comparing study subjects whose dietary habits could be significantly different. Along the same lines, Zarrinpar and colleagues found 6 pairs of discordant twins for NAFLD. The expression of these miRNAs failed to explain this mismatch (34). Therefore, the idea that environmental factors are the main factors involved in modifying the expression of these molecules is quite plausible. This fact has been reported in a review carried out by Ji and colleagues, in which they highlight that the expression level of these molecules is altered in obese and overweight patients (35). Specifically, in the case of miR-122, it has been described how in healthy individuals the liver produces this molecule; however, in conditions of obesity, adipose tissue increases the production and secretion of circulating miR-122 as a mechanism for maintaining hepatic function (35, 36). In the context of HIV infection, our study is contrary to the study carried out by Austermann and colleagues, which is one of the few studies that has analyzed the association of miRNAs with NAFLD in HIV infection (19). This study with a longitudinal design analyzed the role of miR-200a as a possible predictor of NAFLD, as well as a tool for its progression. However, as in the case of studies carried out in the general population, it has some limitations. One of them is the use of inadequate standardization of the results, which the author warns as a limitation of the study. The inclusion of patients with obesity (BMI greater than 30 kg/m^2^) denotes another limitation of the study, since the determination of CAP in these patients may be biased, as the technique is not indicated for these patients (16). Finally, the increased expression of miR-200a is closely linked to being overweight (p<0.01), so it cannot be concluded that this molecule is a useful biomarker to identify patients with NAFLD.

For all these reasons, since the design of this study has allowed the analysis of both study groups with a similar BMI, the results obtained confirm that the alteration of miRNAs reported in various studies in the general population could be more related to other factors such as obesity and diabetes than to the presence of NAFLD. Thus, diet and underlying weight factors would be more important risk factors than epigenetic mechanisms, such as miRNAs, in the development of NAFLD. However, there must be underlying risk factors for HIV infection that must be evaluated to elucidate the high prevalence of NAFLD in this special population compared to the general population. On the other hand, since the study was carried out in PLWH patients, we cannot completely rule out that this absence of differential patterns of miRNAs occurs in the same way in the general population. In fact, the results of this study suggest the existence of a hidden underlying classification for these patients who will be evaluated in future studies, given that the analysis showed a highly homologous profile among 7 patients (5 cases and 2 controls) whose classification was not associated with the degree of NALFD (**Figure 2**).

Our study has three limitations. First, patients were diagnosed with NAFLD through CAP and not using the gold standard technique, which is the histological study of liver biopsy. Second, although patients with an alcohol consumption greater than 20 grams/day were not included, this was collected on a self-reported basis. Last, our study did not include a healthy volunteer group. This aspect is of great interest to assess the possible influence of HIV infection itself on the difference in expression of miRNAs and its relationship with severe NAFLD.

## 5 Conclusions

This study shows that the miRNA transcriptome profile of nonobese patients with severe NAFLD do not differ from the profile of PLWH without NAFLD. Therefore, miRNAs are not ideal markers for the diagnosis of severe NALFD. Further studies are needed to determine other -omic biomarkers that could be linked to the development of severe NAFLD in this special population.

## Data availability statement

The data presented in the study are deposited in public repository (ArrayExpress collection; Biostudies), with code E-MTAB-12974.

## Ethics statement

The studies involving humans were approved by Clinical Trials and Ethics Committee of Hospital Universitario Ntra. Sra. de Valme. The study was approved with code PI18/00606. The studies were conducted in accordance with the local legislation and institutional requirements. The participants provided their written informed consent to participate in this study.

## Author contributions

Conceptualization: FM, R-JA and RA; Data curation: FM, R-JA, MM and RA; Formal Analysis: FM, R-JA, MM and RA; Funding acquisition: RA; Investigation: FM, C-GJ, L-LP, ACG, DC-M, R-CI, C-JM, P-VI and RA; Methodology: FM, R-JA, MM, C-GJ, P-VI and AR; Supervision: R-JA and RA; Validation: FM, R-JA and RA; Visualization: FM, R-JA and RA; Writing-original draft: FM, R-JA and RA; Writing-review & edition: All authors. All authors contributed to the article and approved the submitted version.
