# Supplementary material for: Lack of associations of microRNAs with severe NAFLD in people living with HIV: discovery case-control study
Source: Front Endocrinol (Lausanne). 2023 Sep 22;14:1230046. doi: 10.3389/fendo.2023.1230046 (PMC10556652; doi:10.3389/fendo.2023.1230046)
Supplement: Supplementary file 2 [file DataSheet_1.pdf]

Supplemental data

Figure S1. Graphical representation of statistical process control.

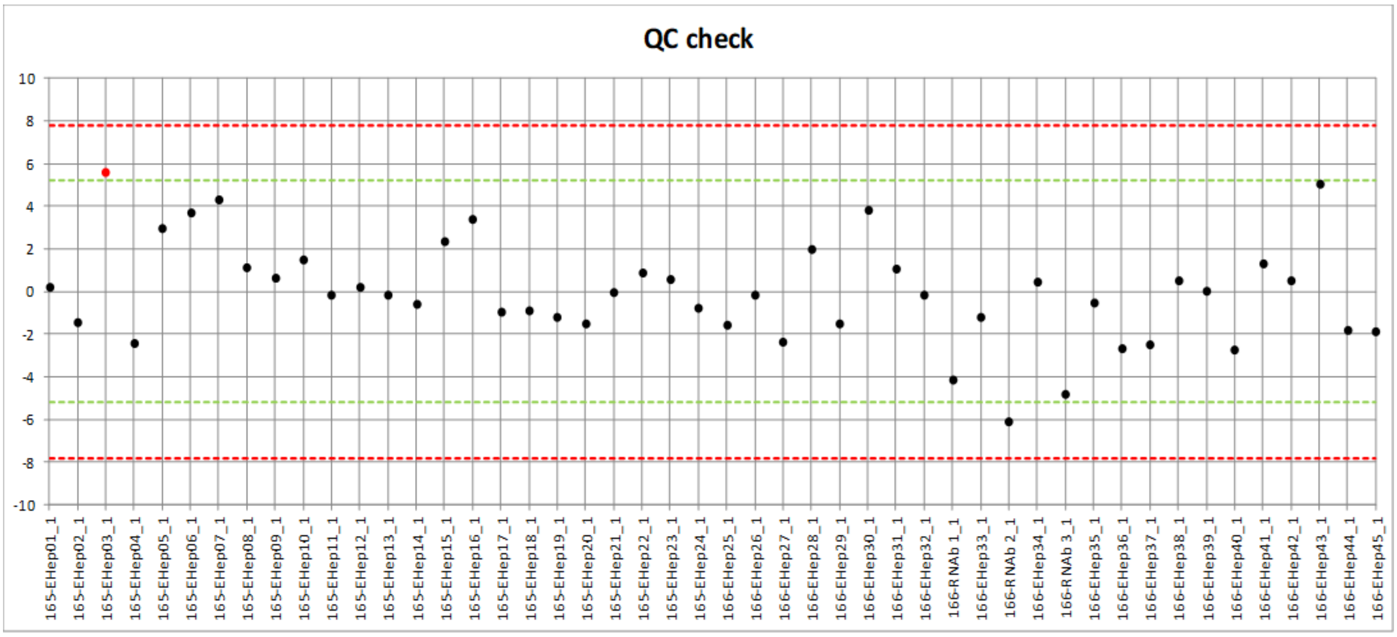

Figure S2. Correlation in RNA signal across the three Reference RNA controls (brain). Pearson correlation is included. * stands for p<0.001.**

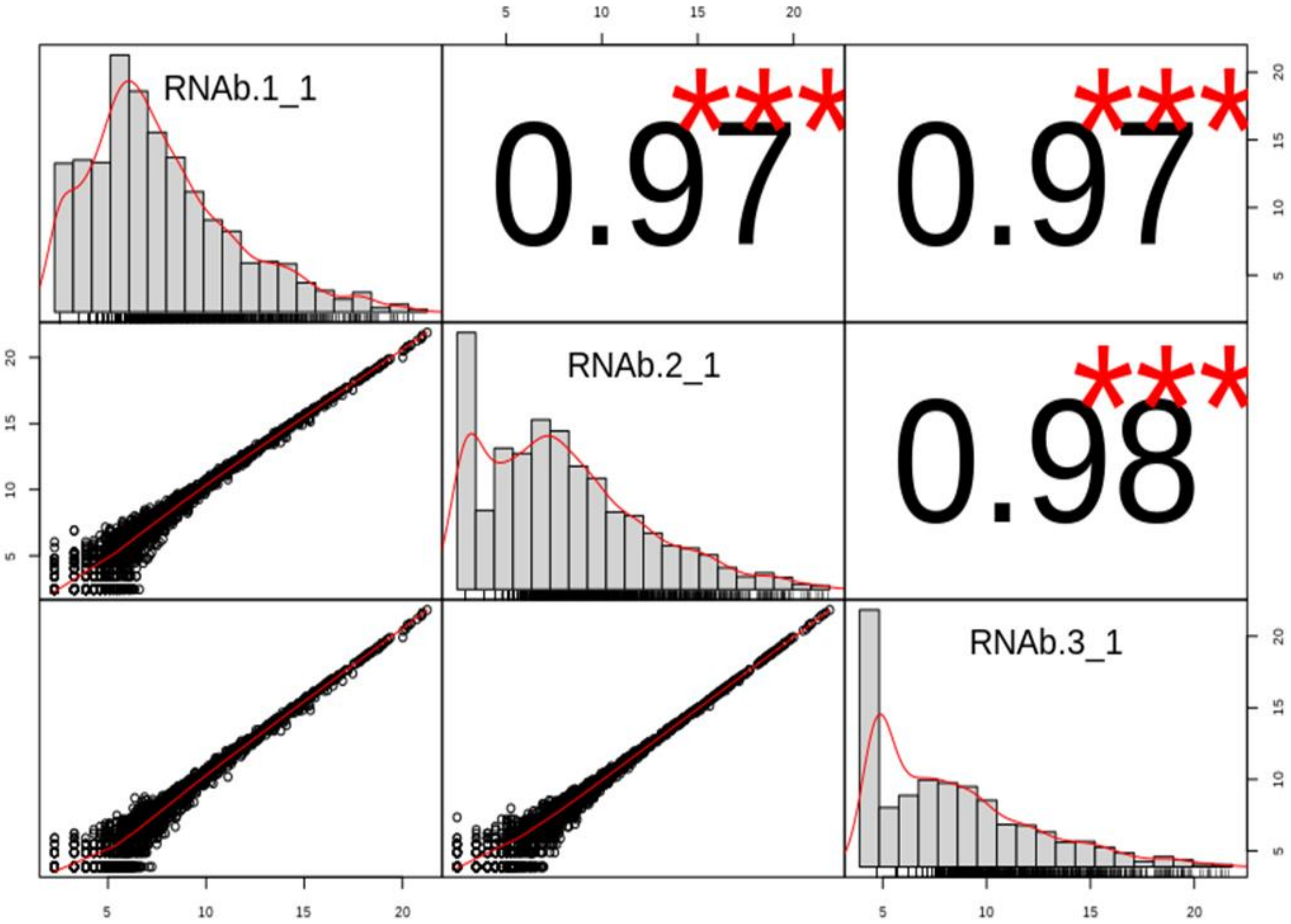

Supplemental data: Excel file with quantification data (raw, QC raw, CPM, and median normalized).
